# Fyn kinase mediates the development of rats with chronic obstructive pulmonary disease by modulating the activation of p38 MAPK and NF-κB

**DOI:** 10.22038/ijbms.2025.82400.17818

**Published:** 2025

**Authors:** Qiangqiang Chu, Yan-bei Zhang, Nan Shen, Song Peng, Yong-xiang Wu, Feng Chu, Jing-cheng Ding

**Affiliations:** 1 Department of Geriatric Respiratory and Critical Care Medicine, The First Affiliated Hospital of Anhui Medical University, Hefei, 230022, Anhui, China; 2 Department of General Practice, The Third Affiliated Hospital of Anhui Medical University, Hefei, 230061, Anhui, China; 3 Bengbu Medical University, Bengbu, 233030, Anhui, China; 4 Department of Critical Care Medicine, The Third Affiliated Hospital of Anhui Medical University, Hefei, 230061, Anhui, China; 5 The Third Affiliated Hospital of Anhui Medical University, Hefei, 230061, Anhui, China

**Keywords:** AZD0530, Chronic obstructive - pulmonary disease, Fyn, MAPK, NF-κB

## Abstract

**Objective(s):**

The current research was conducted to study the function of Fyn in a rat model of chronic obstructive pulmonary disease (COPD).

**Materials and Methods:**

COPD in rats was induced by intratracheal instillation of lipopolysaccharide and long-term exposure to cigarette smoke. Subsequently, the rats were treated with the Fyn-specific inhibitor AZD0530. Pulmonary function, pathological appearance, and inflammatory factors were assessed in rats with COPD.

**Results:**

AZD0530 significantly ameliorated pulmonary function and improved the pathological manifestations of COPD in rats. AZD0530 decreased MCP-1 and CD68 expression in lung tissues, reduced inflammatory cell accumulation, and decreased TNF-α and IL-6 production in bronchoalveolar lavage fluid. In an *in vitro *study, pharmacological inhibition of Fyn or knockdown of Fyn by siRNA inhibited lipopolysaccharide- and cigarette smoke extract-induced TNF-α and IL-6 secretion in the human bronchial epithelial cell line BEAS-2B. Furthermore, inhibition of Fyn by either the inhibitor or siRNA Fyn reduced the phosphorylation of p38 MAPK- and NF-κB-related molecules, which strongly affected the occurrence of inflammatory responses.

**Conclusion:**

Collectively, these data show that Fyn promotes COPD development by modulating the p38 MAPK and NF-κB signaling pathways. Fyn might be a promising therapeutic target for COPD.

## Introduction

Chronic obstructive pulmonary disease (COPD) is a common disease characterized by airflow obstruction and persistent respiratory symptoms, which can further lead to pulmonary heart disease and respiratory failure. The World Health Organization has announced that COPD is the third leading cause of death worldwide, resulting in 3.23 million deaths in 2019. Moreover, COPD has become the third most prevalent chronic disease in China (1). Currently, the recommended pharmacotherapy for patients with mild disease includes β2 agonists and anticholinergic agents, and for patients with acute exacerbation of COPD, corticosteroids, antibiotics, and oxygen therapy are administered. Although these therapies effectively improve symptoms, no treatment can suppress or reverse disease progression. As a result, a strategy for preventing the progression of COPD is currently unavailable (2-5). Therefore, it is necessary to identify potential targets to treat the development of COPD.

The pathophysiological mechanism underlying COPD has not been fully elucidated. Several factors, such as genetics, sex, occupation, airway hyperresponsiveness, and infection, have been disclosed as important factors for developing COPD (6). Chronic inflammatory response is a significant factor involved in promoting COPD progression. During the development of COPD, numerous neutrophils accumulate in the airways of COPD patients, and these cells secrete serine proteases, leading to airway remodeling (7). Moreover, the activation of macrophages regulates tissue inflammation (6) through the secretion of many proinflammatory cytokines, such as TNF-α, IL-1β, and IL-6 (6). Tyrosine kinase Fyn is a member of the Src kinase family. Fyn kinases perform various cell biological functions that affect cell growth, survival, adhesion, and cytoskeletal remodeling (8). Additionally, Fyn participates in repairing tissue injury and inflammatory responses. In a previous study, pharmacological inhibition of Fyn in mast cells was shown to prevent Type I hypersensitivity in mice (9). Moreover, Fyn has been revealed to mediate tissue inflammation in an animal model of endotoxemia (10). Interestingly, Fyn activation is involved in the hyperreactivity of bronchial smooth muscle in rats caused by angiotensin II (11). Therefore, in this study, we aimed to investigate the function of Fyn in the development of COPD.

## Materials and Methods

### Animals, COPD induction and treatment

Forty-eight Wistar rats weighing 180±20 g were commercially purchased from the SLAC laboratory animal CO. LTD (Shanghai, China) and bred in the experimental animal center of Anhui Medical University (Hefei, China). The rats were housed under pathogen-free conditions with a 12 h:12 hr light:dark cycle and had free access to food and water.

The rats were exposed to cigarette smoke daily, and lipopolysaccharide (LPS, Sigma‒Aldrich Co. St. Louis, MO, USA) was instilled into the trachea to induce COPD (12, 13). In brief, 10% chloral hydrate was used to anesthetize the animals, and then, LPS (200 μg/100 μl) was administered to the rats on Days 1 and 14 via intratracheal instillation. The rats were subsequently maintained in a cigarette smoke environment that was established by placing 12 cigarettes in a chamber (30 cm×30 cm×60 cm) for 30 min once a day for 28 days, except for the 1st and 15th days. Six groups were established: a control group, a COPD group, a Fyn inhibitor group (AZ0530 5.0–20.0 mg/kg/day, Aladdin Reagent Co. Ltd., Shanghai, China), and a Dexamethasone group (DEX, 2 mg/kg/day, Sigma‒Aldrich Co. St. Louis, MO, USA) (n=8/group). AZ0530 and DEX were orally administered to the rats every day from Day 15 to Day 28, whereas saline was administered to the rats in the control normal and COPD groups.

### Measurement of pulmonary function

Pulmonary function was observed at the end of the study (14, 15). In brief, the trachea of anesthetized rats was cannulated and connected to an instrument (Buxco Inc., Wilmington, NC, USA). The functional residual capacity (FRC), forced expiratory volume in 100 ms (FEV100), forced vital capacity (FVC), maximum mid-expiratory flow (MMEF), and peak expiratory flow (PEF) were used to evaluate airflow limitations and recorded.

### Collection of bronchoalveolar lavage fluid (BALF)

BALF was collected from the animals. In brief, at the end of the study, 3 ml of sterile phosphate-buffered saline (PBS) was instilled into the trachea and then collected. The process was repeated 3 times to collect the BALF. The fluid was subsequently centrifuged at 2000 rpm for 10 min at 4 °C. The levels of proinflammatory cytokines in the collected supernatant were collected. Moreover, the total number of inflammatory cells in the fluid was determined via a hemocytometer. Neutrophil counts were conducted on slides stained with Wright‒Giemsa (Beyotime Biotechnology, Shanghai, China), and the percentage of neutrophils was calculated.

### Histopathology evaluation

Lung tissues were fixed in 4% paraformaldehyde for 24 hr. Then, 4-µm-thick sections were cut and stained with H&E. Pathological manifestations in the lungs were semiquantitatively evaluated and analyzed using Image-Pro Plus 6.0 software (Media Cybernetics, Rockville, USA) based on a previously published report. The indicators of airway remodeling include collagen thickness, smooth muscle thickness, the ratio of wall thickness/bronchiole diameter, and the ratio of wall area/total bronchiole area.

### Immunohistochemical assay

Lung tissue sections were prepared. The sections were covered with 3% BSA for 30 min and incubated with anti-MCP-1 or anti-CD68 antibodies (Abcam, Cambridge, MA, USA) in DAB solution. An Olympus light microscope was used to observe positive expression, and then semiquantitative analysis was performed using Image-Pro Plus 6.0 software (Media Cybernetics, Rockville, USA).

### CSE preparation

Cigarette smoke extract (CSE) was produced following a previously published method (16). In brief, one cigarette without a filter (Huangshan Brand; China Tobacco Anhui Industrial Co. LTD) was aspirated into a vacuum pump. The smoke was drawn into 50 ml of PBS for three minutes. The pH value of CSE was adjusted to 7.4, and CSE was sterilized through a 0.22-μm filter. CSE (100%) was diluted to a 10% concentration with culture medium and used within 30 min of preparation.

### Cell culture and treatment

Human bronchial epithelial cells (BEAS-2B) were commercially acquired from the American Type Culture Collection (Rockville, MD, USA) and cultured in Dulbecco’s modified Eagle’s medium supplemented with 10% fetal bovine serum and 1% penicillin/streptomycin at 37 °C in an incubator with 5% CO_2_. BEAS-2B cells were pretreated with different concentrations of AZD0530 for two hours and then stimulated with 10% CSE for 15 min to mimic the smoking of one cigarette. After that, the CSE was removed, and the cells were washed with PBS, cultured in fresh medium, and treated with LPS (100 ng/ml, Sigma‒Aldrich, USA) for 24 hr.

### siRNA Fyn transfection

Fyn siRNAs were designed and synthesized by GenePharma Co., Ltd. (Shanghai, China). The sequences of the Fyn siRNAs that were used were as follows: Fyn siRNA-1# Sense: GGAUAAAGAAGCAGCGAAAdTd T; Anti-sense: UUUCGCUGCUUCUUUAUCCdTdT; Fyn siRNA-2# Sense: GGUUCACAAUCAA GUCUGA dTdT; Anti-sense: UCAGACUUGAUUGUGAACCd TdT; Fyn siRNA-3# Sense: AGUAG UUCCCUGUCACAAAdTdT; Anti-sense: UUUGUG ACAGGGAACUACUdTdT; Negative Control (NC) Sense: UUCUCCGA ACGUG UCA CGUdTdT; Anti-sense:ACGUGACACGUUCGGAGAAdTdT. The cells cultured in 24-well plates were transfected with 40 nM siRNAs via Lipofectamine 2000 (Invitrogen, Carlsbad, CA, USA). Western blotting was performed to validate the efficiency of Fyn knockdown. 

### Measurement of inflammatory cytokines

Proinflammatory cytokine levels in the BALF supernatants and the cultured cells were measured using ELISA kits (Beyotime, Shanghai, China).

### Western blotting

Whole and nuclear proteins were extracted from tissues and cells with a lysis buffer (Beyotime, Shanghai, China) and a nuclear protein extraction kit (Beyotime, Shanghai, China). The protein samples were separated by SDS‒PAGE and transferred to PVDF membranes (Millipore, Burlington, MA, USA). After routine operation, the membranes were incubated with primary antibodies against Fyn (Abcam, Cambridge, MA, USA), p38 (Abcam, Cambridge, MA, USA), Iκba (Abcam, Cambridge, MA, USA), p65 (Abcam, Cambridge, MA, USA), p-p38 (Abcam, Cambridge, MA, USA), p-Iκba (Abcam, Cambridge, MA, USA) and p-p65 (Abcam, Cambridge, MA, USA) overnight at 4 °C, followed by incubation with appropriate secondary antibodies for one hour at room temperature. The protein blots were visualized using enhanced chemiluminescence, and the image density was analyzed using Image-Pro Plus 6.0 (Media Cybernetics, Inc., Bethesda, MD, USA).

### Statistical analysis

All data are presented as the means ± standard deviations (SDs). Statistical analysis was conducted using SPSS Statistics 18.0 software (SPSS Inc., Chicago, IL, USA). One-way ANOVA and subsequent Tukey’s *post hoc* analysis were applied to compare the differences. A statistically significant difference was confirmed when *P*<0.05.

## Results

### Fyn inhibitor AZD0530 improves the pulmonary function of COPD model rats

**Figure 1:**
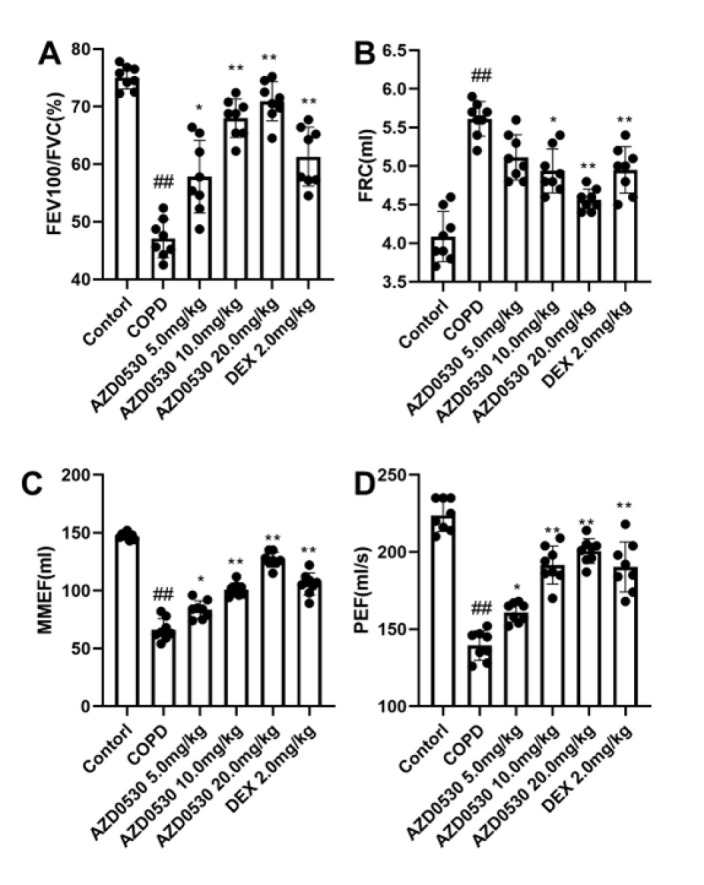
Fyn inhibitor AZD0530 ameliorated the pulmonary function of rats with COPD

We tested the lung function of COPD model rats. As shown in Figure 1, LPS stimulation and chronic exposure to cigarettes decreased the values of FEV100/FVC, MMEF, and PEF and increased the value of FRC at the end of the study (*P*<0.01), indicating decreased lung function in the model rats. However, the impaired pulmonary function caused by LPS and chronic exposure to cigarettes was abolished by treatment with the Fyn inhibitor AZD0530 (*P*<0.05).

### Fyn inhibitor AZD0530 improves pathological manifestations in COPD model rats

**Figure 2:**
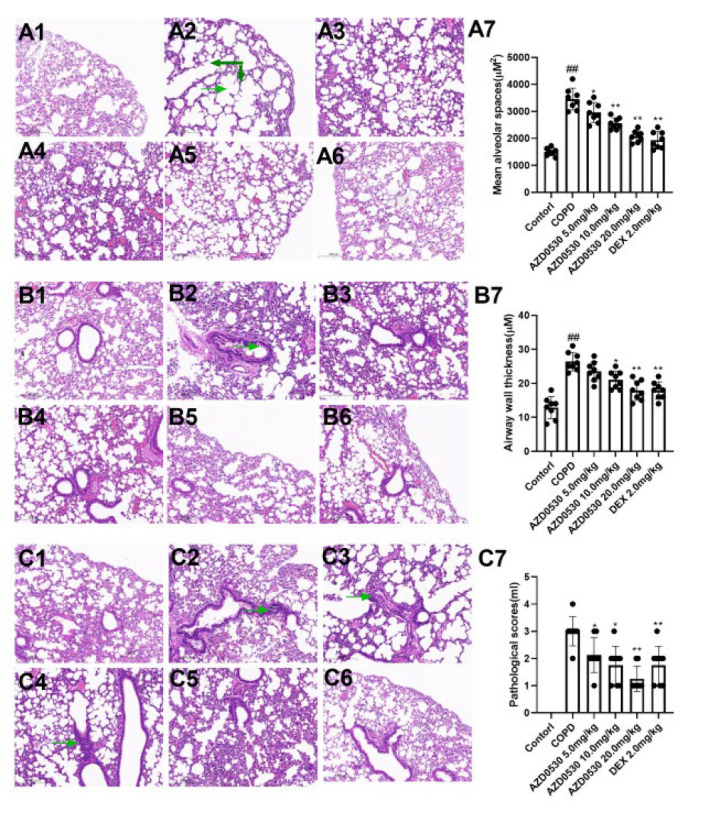
Fyn inhibitor AZD0530 ameliorates pathological manifestations in the lung tissue of COPD model rats

As shown in Figure 2, LPS stimulation and chronic cigarette exposure caused significant alveolar dilation, damage to the alveolar wall, fusion of the alveolar wall, thickening of the bronchial walls, disordered epithelial cells, and extensive infiltration of inflammatory cells. However, in the rats treated with AZD0530 or the positive control drug, the degree of pathological damage and appearance clearly improved (*P*<0.05).

### Fyn inhibitor AZD0530 inhibits the inflammatory response in COPD model rats

**Figure 3:**
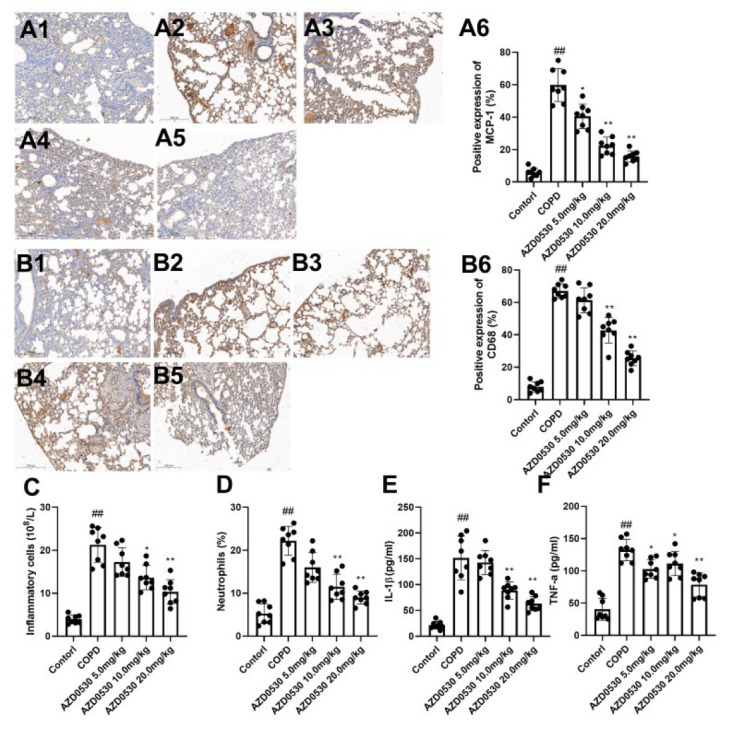
Fyn inhibitor AZD0530 inhibited the pulmonary inflammatory response in COPD model rats

To assess the effects of AZD0530 on inflammatory responses in the lung tissues of COPD model rats, we detected and analyzed macrophage infiltration in the lung tissues, inflammatory cell populations, and proinflammatory cytokine levels in the BALF. As shown in Figure 3A1-3A6, the positive expression of MCP-1 in the COPD group was markedly greater than that in the control group (*P*<0.01). Moreover, CD68 expression in the COPD group was markedly increased compared with that in the control group, suggesting increased infiltration of macrophages in the COPD group (Figure 3B1-3B6, *P*<0.01). However, oral AZD0530 administration significantly reduced MCP-1 and CD68 expression in COPD model rats (*P*<0.05). Furthermore, compared with the control group, the COPD group presented increased total inflammatory cell and neutrophil counts and increased IL-1β and TNF-α levels in the BALF (*P*<0.01). Oral administration of AZD0530 at dosages of 10 mg/kg and 20 mg/kg effectively reduced the number of inflammatory cells, the percentage of neutrophils, and the levels of IL-1β and TNF-α in COPD model rats (Figure 3C-3F, *P*<0.05). However, we found that treatment with AZD0530 at a dosage of 5 mg/kg did not ameliorate inflammatory cells, neutrophils, or IL-1β levels, indicating a dose-dependent anti-COPD effect of AZD0530. Our data suggest that AZD0530 attenuates pulmonary inflammation in COPD model rats.

### AZD0530 inhibits the activation of p38 MAPK/NF-κB p65 in COPD model rats

**Figure 4:**
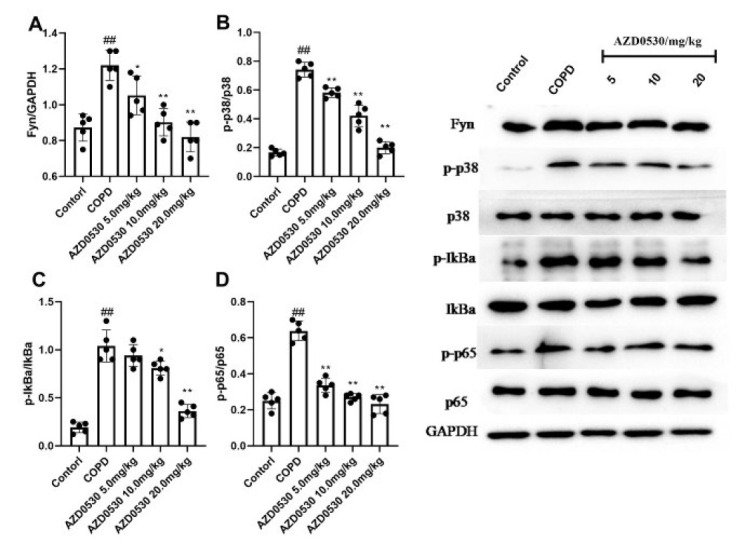
AZD0530 inhibited the activation of the p38MAPK/NF-κB p65 axis in COPD model rats

The p38MAPK/NF-κB p65 signaling axis is pivotal in initiating and amplifying inflammation in COPD patients (17). Therefore, we measured inflammatory reaction-related proteins in lung tissues by western blotting. Figure 4 shows that Fyn expression was dramatically greater in the COPD group than in the control group (*P*<0.01). However, the Fyn level was lower in the AZD0530 group than in the COPD group (*P*<0.05). Further analysis revealed that the p38MAPK/NF-κB p65 axis was more highly activated in COPD model animals than in control animals (*P*<0.01), as reflected by increased phosphorylation of p-p38, p-Iκba, and p-p65. Moreover, we observed that AZD0530 reduced the expression of p-p38, p-Iκba, and p-p65 (*P*<0.05) without influencing the total expression of p38, Iκba, or p65 in COPD model rats.

### AZD0530 inhibits LPS- and CSE-induced inflammatory cytokine secretion by BEAS-2B cells

**Figure 5:**
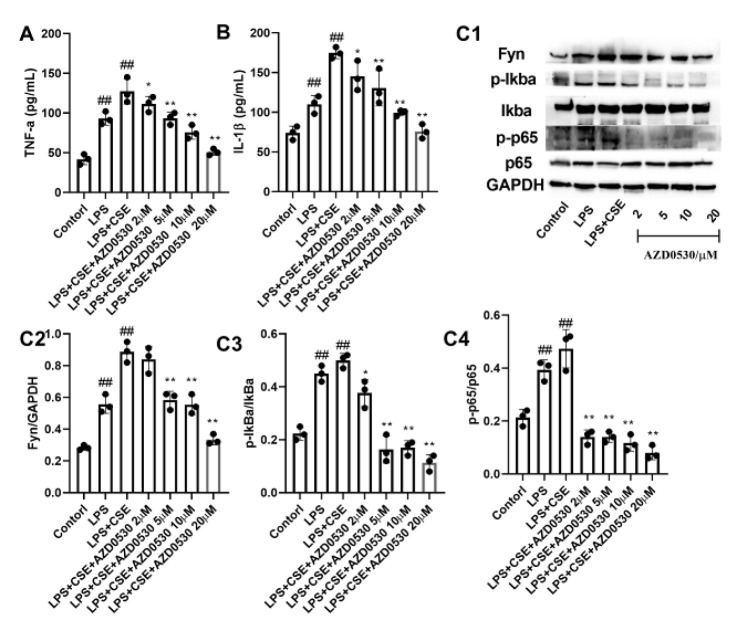
AZD0530 inhibited LPS- and CSE-induced secretion of proinflammatory cytokines by Beas-2B cells

To further elucidate the role of Fyn in airway inflammation, BEAS-2B cells were stimulated with LPS and CSE, and the resulting TNF-α and IL-1β levels were investigated. As shown in Figure 5, LPS and CSE stimulation markedly increased the expression of TNF-α and IL-1β (*P*<0.01). However, intervention with the Fyn inhibitor AZD0530 resulted in a significant and concentration-dependent decrease in the expression of TNF-α and IL-6, with 20 μM AZD0530 having the most significant effect (*P*<0.05). Moreover, Fyn, p-p38, p-Iκba, and p-p65 expression was markedly increased in cells stimulated with LPS and CSE compared with those in the LPS and CSE groups. As expected, the effects caused by LPS and CSE were significantly abolished after pre-treatment with AZD0530 (*P*<0.05).

### Knockdown of Fyn abolishes the LPS- and CSE-induced increase in the production of TNF-α and IL-1β

**Figure 6:**
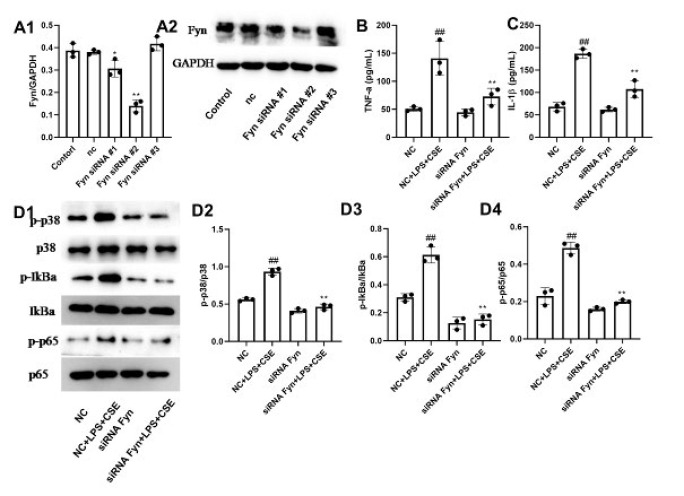
Knockdown of Fyn decreased the production of proinflammatory cytokines and inhibited the activation of the p38 MAPK/p65 axis in LPS- and CSE-treated Beas-2B cells

We silenced Fyn expression by siRNA transfection. As shown in Figure 6A1-6A2, Fyn was successfully knocked down with siRNA#2, resulting in the best silencing efficiency (*P*<0.01). As shown in Figure 6B-6C, LPS and CSE stimulation increased the secretion of TNF-α and IL-1β and activated p38, Iκba, and p65 (*P*<0.01). Interestingly, the proinflammatory effect of costimulation was significantly abolished in Fyn-knockdown cells (*P*<0.01). Finally, we found that Fyn silencing could prevent LPS- and CSE-induced activation of p38, Iκba, and p65 (Figure 6, *P*<0.01).

## Discussion

Research has demonstrated that Fyn performs pivotal functions in various respiratory diseases. For example, the inhibition of Fyn by PP2 mitigated acute lung injury (18). Moreover, Fyn kinase has been shown to regulate endothelial barrier dysfunction in *Plasmodium berghei-*infected mice (19). However, little is known about the function of Fyn in COPD. To further test this hypothesis, we pharmacologically inhibited Fyn in COPD model rats and then evaluated the COPD symptoms. AZD0530 was initially developed as an antineoplastic drug (20). Recently, AZD0530 has been developed as a Fyn inhibitor in the treatment of Alzheimer’s disease (21). AZD0530 has numerous desirable properties. First, AZD0530 has a good inhibitory effect on Fyn within the low nM range (22), and second, clinical trials have shown that AZD0530 is safe and well tolerated in patients with Alzheimer’s disease or tumors (23,24). Therefore, AZD0530 was used in the present study. During the development of COPD, damaged lungs exhibit airflow obstruction, which may result from the narrowing of small conducting airways, loss of lung elastic recoil, or both. Therefore, the diagnosis of airflow obstruction has been determined by spirometry and is extensively reflected by changes in the parameters of peak expiratory flow, maximal mid-expiratory flow, and increased functional residual capacity. In the present study, LPS stimulation and chronic exposure to cigarette smoke decreased the values of FEV100/FVC, MMEF, and PEF. They increased the value of FRC (*P*<0.01), indicating decreased respiratory function and potential airflow obstruction in model rats. However, the injury caused by LPS and chronic exposure to cigarettes was abolished by treatment with the Fyn inhibitor AZD0530 (*P*<0.05). Moreover, AZD0530 inhibited pathological changes in COPD model rats, indicating that AZD0530 is a promising anti-COPD drug. To the best of our knowledge, this is the first report to reveal the effectiveness of a Fyn inhibitor on the severity of COPD in an animal model.

Inflammation is considered a double-edged sword. The inflammatory response is a defensive reaction to infection or other injuries. However, excessive tissue inflammation has been identified as a risk factor for the progression of many diseases. Inflammation promotes the development of COPD (25). MCP-1 is a proinflammatory chemokine that plays a key role in recruiting monocytes to sites of injury and infection. MCP-1 can also recruit macrophages, neutrophils, and lymphocyte inflammatory cells to the lungs, thereby increasing proinflammatory cytokine secretion, tissue inflammation, and COPD disease severity (26). Briefly, neutrophils and macrophages mediate oxidative stress and participate in airway remodeling in airways affected by COPD. On the other hand, oxidative stress facilitates the accumulation of numerous neutrophils in the airways of COPD patients. Moreover, the activation of macrophages regulates inflammation via the production of many cytokines that mediate the development of COPD (27-28). TNF-α stimulation damages the alveolar epithelium by mediating endothelial adhesion molecules and the accumulation of polymorphonuclear leukocytes. Moreover, TNF-α activates an inflammatory cascade together with IL-1β (29). In several previous studies, Fyn has been reported to regulate the inflammatory response. LPS causes increased serum TNF-α and IL-6 levels, which were inhibited in Fyn KO but not in wild-type mice (30). Furthermore, inhibiting macrophage infiltration by improving the redox state requires Fyn-dependent Nrf2 activation (31). In the present study, AZD0530 decreased MCP-1 and CD68 expression in lung tissues, indicating decreased infiltration of macrophages by treatment with a Fyn inhibitor. Moreover, AZD0530 reduced the number of inflammatory cells and the IL-1β and TNF-α levels *in vivo* and *in vitro*.

To better understand the underlying mechanism of AZD0530 efficacy, we investigated the p38MAPK/p65 NF-κB pathway. The main reason is that extracellular stimuli, such as LPS and the TNF receptor, may increase Fyn, causing the activation of Fyn downstream of p38 MAPK and p65 NF-κB (32). As a result, NF-κB activation promotes the transcription of many inflammatory cytokine genes (such as TNF-α, IL-1β, IL-6, and IL-12), thereby exacerbating inflammation. NF-κB is also a transcription factor of M1 macrophages (33, 34). In the present study, we found that p38, Iκba, and p65 levels were unchanged in COPD model rats or CSE-treated cells compared with those of the control groups. However, the phosphorylated p38, Iκba, and p65 levels were increased both *in vivo *and *in vitro*. Normally, NF-κB exists in an inactive form, and its p65 subunit is sequestered in the cytoplasm by the inhibitory unit Iκba. When the IKK complex phosphorylates Iκba, it is ubiquitinated and degraded, releasing p65 to enter the nucleus (35). To reveal the role of Fyn in the development of COPD, AZD0530 and siRNA were both applied in order to inhibit Fyn’s biological action. The main difference between the AZD0530 and siRNA assays is that the siRNA temporarily but specifically reduces the expression of Fyn. At the same time, AZD0530 reduces the function of Fyn and down-regulates its expression in a nonspecific manner (36). In the present study, AZD0530 reduced p-p38, p-Iκba, and p-p65 expression without affecting the total expression of p38, Iκba, or p65. These data suggest that inhibition of Fyn blocks the activation of downstream p38 and p65, thereby terminating the lung inflammatory response. Moreover, further investigations were conducted to verify the function of Fyn in the secretion of TNF-α and IL-1β in COPD, and we confirmed that silencing Fyn effectively suppressed the secretion of TNF-α and IL-1β in epithelial BEAS-2B cells stimulated with LPS and CSE.

The rats were challenged with cigarette smoke and LPS for 28 days in the presence or absence of AZD0530. (A) FEV100/FVC%, (B) FRC, (C) MMEF, and (D) PEF were measured to evaluate the pulmonary function of the animals. The data are expressed as the mean±SD (n=8). ##*P*<0.01, compared with the control group; *P<0.05, **P<0.01, compared with the COPD group.

COPD: Chronic obstructive pulmonary disease; DEX: Dexamethasone; PEF: Peak expiratory flow; MMEF: Maximum mid expiratory flow

The tissues were subjected to H&E staining. Representative histopathological sections of tissues showed a significant appearance of the mean alveolar area in A (right arrow: alveolar dilation; left arrow: fusion of the alveolar wall; downwards arrow: damaged alveolar wall), incrassate bronchial walls in B (right arrow: thickened bronchial walls), and infiltrative inflammatory cell in C (right arrow: extensive infiltrated inflammatory cells). The data are expressed as the mean±SD (n=8). ##*P*<0.01, compared with the control group; *P<0.05, **P<0.01, compared with the COPD group. A1, B1, C1: Control group; A2, B2, C2: COPD; A3, B3, C3: AZD0530 5.0 mg/kg; A4, B4, C4: AZD0530 10.0 mg/kg; A5, B5, C5: AZD0530 20.0 mg/kg; A6, B6, C6: DEX. The results of the quantitative analysis of the pathological manifestations are shown in A7, B7, and C7.

COPD: Chronic obstructive pulmonary disease; DEX: Dexamethasone

The slides of lung tissues were stained with anti-MCP-1 and anti-CD68 antibodies. (A) MCP-1-positive cells; (B) CD68-positive cells; A1, B1: Control group; A2, B2: COPD group; A3, B3: AZD0530, 5.0 mg/kg group; A4, B4: AZD0530, 10.0 mg/kg group; A5, B5: AZD0530, 20.0 mg/kg group. The semiquantitative analysis results are shown for A6 and B6. BALF was collected from rats challenged with cigarette smoke and LPS for 28 days; (C) BALF inflammatory cells; (D) neutrophils; (E) IL-1β; and (F) TNF-α. The data are expressed as the mean±SD (n=8). ##*P*<0.01, compared with the control group; *P<0.05, **P<0.01, compared with the COPD group.

COPD: Chronic obstructive pulmonary disease

Lung tissues were prepared, and protein samples were extracted. The levels of the target proteins were assayed by western blotting. (A) Fyn, (B) p-p38, (C) p-Iκba, and (D) p-p65. The data are expressed as the mean±SD (n=5). ##*P*<0.01, compared with the control group; *P<0.05, **P<0.01, compared with the COPD group.

COPD: Chronic obstructive pulmonary disease

Beas-2B cells were pretreated with different concentrations of AZD0530 for two hours and then exposed to 10% CSE and LPS for 24 hr. A-B: Commercial ELISA kits were used to measure the levels of TNF-α and IL-1β. C1-C4: Samples were extracted from the cells, and the targeted protein levels were assayed by western blotting. The data are expressed as the mean±SD (n=3). ##*P*<0.05, compared with the control group; *P<0.05, **P<0.01, compared with the LPS+CSE group.

LPS: Lipopolysaccharide; CSE: Cigarette smoke extract; Beas-2B: Human bronchial epithelial cells

Beas-2B cells with Fyn knockdown were pretreated with AZD0530 for two hours and then stimulated with 10% CSE and LPS for 24 hr. A: Fyn was effectively silenced by siRNA transfection; B-C: Commercially available ELISA kits were used to measure the levels of TNF-α and IL-1β; D1-D4: The levels of targeted proteins were assayed by western blotting. The data are expressed as the mean±SD (n=3). ##*P*<0.05, compared with the NC group; **P<0.01, compared with NC+LPS+CSE.

LPS: Lipopolysaccharide; CSE: Cigarette smoke extract; Beas-2B: Human bronchial epithelial cells

## Conclusion

Fyn is abnormally expressed in COPD patients. Fyn participates in the pathophysiological progression of COPD by meditating the activation of p38 MAPK and p65 NF-κB. The Fyn inhibitor AZD0530 has good therapeutic effects on COPD model rats.

## Data Availability

The data are available from the corresponding author upon reasonable request.

## Declaration

We have not used any AI tools or technologies to prepare this manuscript.
